# Overdiagnosis of Papillary Thyroid Cancer

**DOI:** 10.1001/jamanetworkopen.2025.59852

**Published:** 2026-02-24

**Authors:** David O. Francis, Louise Davies, Yichi Zhang, Natalia Arroyo, Sara Fernandes-Taylor, Peter Nordby, Benjamin A. Y. Cher, Manasa Venkatesh, Erin J. Aiello Bowles, Oguzhan Alagoz

**Affiliations:** 1 Department of Otolaryngology Head & Neck Surgery, University of Wisconsin-Madison School of Medicine and Public Health, Madison; 2 Wisconsin Surgical Outcomes Research Program, Department of Surgery, University of Wisconsin-Madison School of Medicine and Public Health, Madison; 3 Department of Surgery, University of Wisconsin-Madison School of Medicine and Public Health, Madison; 4 Department of Industrial and Systems Engineering, College of Engineering, University of Wisconsin-Madison, Madison; 5 University of Wisconsin Carbone Cancer Center, University of Wisconsin-Madison School of Medicine and Public Health, Madison; 6 Division of Geriatrics and Gerontology, Department of Medicine, University of Wisconsin-Madison School of Medicine and Public Health, Madison; 7 Kaiser Permanente Washington Health Research Institute, Kaiser Permanente Washington, Seattle

## Abstract

**Importance:**

The magnitude of thyroid cancer overdiagnosis in the US, accounting for the true increase in underlying disease, has not been quantified. The implications of reducing thyroid ultrasonography use for these rates has not been calculated.

**Objective:**

To estimate US population-level rates of papillary thyroid cancer (PTC) overdiagnosis, and to identify the implications of reducing thyroid ultrasonography use for thyroid cancer incidence and overall mortality.

**Design, Setting, and Participants:**

This study used a validated simulation model (Papillary Thyroid Carcinoma Microsimulation Model) of contemporary PTC incidence in the adult US population between 1991 and 2019. Data analysis was conducted from June 2024 to August 2025.

**Exposures:**

Thyroid cancer incidence and referrals for ultrasonography of nonpalpable thyroid nodules.

**Main Outcomes and Measures:**

Model-estimated relative and absolute rates of PTC overdiagnosis, stratified by sex. The model estimated changes in the rates of thyroid cancer incidence and overall mortality if ultrasonography rates were reduced.

**Results:**

The simulation included all adults, aged 18 years or older, in the US. Between 1991 and 2019, the model estimated that 72% to 94% of PTC cases were overdiagnosed. While the proportion of overdiagnosis was slightly higher for women compared with men (75%-95% vs 63%-90%), the absolute rate of overdiagnosis was higher among women compared with men (13-17 per 100 000 individuals vs 3-5 per 100 000 individuals). This rate translated into 443 212 to 573 705 women and 107 804 to 154 504 men who were overdiagnosed with PTC over 28 years. Reducing use of thyroid ultrasonography for nonpalpable nodules by 33% and 67% would have decreased PTC incidence by 17% (18 to 15 per 100 000 individuals) and 41% (18 to 11 per 100 000 individuals) in 2019, respectively, a less than 0.1% change in overall mortality.

**Conclusions and Relevance:**

This study found that thyroid cancer overdiagnosis in the US remains substantial, even after accounting for possible increases in true incidence. This finding suggests an opportunity to reduce unnecessary thyroid ultrasonography referrals, particularly for nonpalpable nodules, and to reduce unnecessary diagnoses and treatment-related harms without increasing mortality.

## Key Points

**Question:**

What proportion of papillary thyroid cancer (PTC) cases were overdiagnosed between 1991 and 2019, and what might be the implications of reducing thyroid ultrasonography use for PTC incidence and overall mortality in the US?

**Findings:**

In this study, a simulation model that accounted for changes in incidence during the study period estimated that between 72% and 94% of PTC cases were overdiagnosed in the US between 1991 and 2019. Reducing thyroid ultrasonography for nonpalpable nodules would decrease PTC incidence, with a negligible change in overall mortality.

**Meaning:**

The finding that thyroid cancer overdiagnosis remained substantial despite accounting for a true increase in incidence suggests that reducing ultrasonography referrals for nonpalpable nodules could help mitigate overdiagnosis.

## Teaser

This study uses a validated mathematical simulation model to estimate the overdiagnosis rate of papillary thyroid cancer from 1991 through 2019 in the US.

## Introduction

In the US, thyroid cancer incidence has increased 250% since 1990—primarily due to the detection of small, early-stage papillary thyroid cancer (PTC)—without any improvement in mortality, a problem mirrored in many countries around the world.^1,2^ A large subclinical reservoir of thyroid cancer has long been known to exist, as evidenced by autopsy studies showing that approximately 10% of both men and women have small PTCs and die without ever knowing they were there.^3,4,5^ The pattern of increasing incidence, stable mortality, and a known subclinical reservoir is consistent with the well-established epidemiological phenomenon of overdiagnosis^6,7,8^: the detection and treatment of cancers with no benefit to population mortality. This phenomenon has also been observed in prostate cancer,^9,10,11,12,13^ melanoma,^14,15,16,17^ and ductal carcinoma in situ of the breast.^18,19,20^ However, to our knowledge, no studies have quantified the magnitude of overdiagnosis in thyroid cancer in the US using data that account for current clinical practice and epidemiological patterns.

Ultrasonography referral initiates the diagnostic pathway to thyroid cancer, and there are no official guidelines on when to refer patients for an ultrasonography. People are referred for ultrasonography for symptoms, physical examination findings, or after incidental nodules are discovered on cross-sectional imaging performed for other reasons. The overdiagnosis problem is urgent and getting worse over time. Since 2000, the rates of thyroid ultrasonography and cross-sectional imaging studies in the US have increased more than 5-fold and 10-fold,^21^ respectively. Detection of nodules necessarily leads to biopsies and has led to increased thyroid cancer incidence.^22^

Once diagnosed, the overwhelming majority of patients undergo surgical removal of the cancer, which carries attendant adverse effects and complication risks and may potentially diminish quality of life.^23^ This clinical momentum is a problem because some surgeries are unnecessary. Instead, cancers can be monitored for clinical activity and removed only when or if they grow.^24,25,26^ Even better, strategies could be devised to avoid unnecessary cancer detection from the outset. However, before clinicians and policymakers can devise strategies to reduce thyroid cancer overdiagnosis, it is first necessary to quantify the magnitude of the problem. In so doing, it is important to account for a potential concomitant true increase in the biological risk of developing thyroid cancer due to new or emerging risk factors, such as the rising incidence of obesity.^27^ In this study, we aimed to estimate US population-level rates of PTC overdiagnosis from 1991 and 2019 using a validated mathematical model that accounts for a true increase in disease. We also sought to estimate the implications of reducing ultrasonography use on thyroid cancer incidence and overall mortality to support policy and guideline development efforts to decrease overdetection and overtreatment of PTC.

## Methods

### Model Description

This study used the previously validated Papillary Thyroid Carcinoma Microsimulation Model (PATCAM). The model, which is summarized here but is described in detail in a previous publication,^28^ is informed by high-quality data sources (eTable 1 in Supplement 1). Cancer microsimulation models have been successfully applied as key epidemiological tools to support clinicians and policymakers, including the US Preventive Services Task Force, in addressing critical problems in cancer control.^29,30,31,32^ The University of Wisconsin-Madison Minimal Risk Institutional Review Board deemed this simulation model exempt from ethics review and informed consent requirement because it was secondary research.

PATCAM replicates US sex- and age-specific PTC incidence, stage distribution, and mortality between 1975 and 2019 as reported by the National Cancer Institute Surveillance, Epidemiology, and End Results (SEER) program, the national standard for population-based incidence estimates.^33^ From a modeling standpoint, retaining the full historical window was necessary because replicating the increase, peak, and subsequent stabilization of PTC incidence was central to calibrating the natural history of disease. Restricting calibration risked omitting the period during which the epidemiological shifts in detection and diagnosis occurred, thereby reducing the ability of the model to distinguish true changes in disease burden from changes in diagnostic intensity. The model has been externally validated against 3 active surveillance studies of PTC (1 from Japan, the largest in the world, and 2 from the US).^26,34,35^ Specifically, PATCAM accurately estimated the reported rates of tumors, which grew more than 3 to 5 mm over 5 and 10 years, from these studies.^26,34,35^

### Papillary Thyroid Cancer Detection

The model represents 2 major diagnostic pathways for individuals to receive thyroid ultrasonography: a referral for palpable nodules (palpable pathway) or a referral for other indications (nonpalpable pathway). The nonpalpable pathway includes a referral for symptoms the patient or clinician attributed to the thyroid gland, such as globus sensation or hoarseness, and for follow-up of incidental thyroid nodules detected on cross-sectional imaging.

The model represents the probability that an individual is referred for ultrasonography through an age- and sex-specific function of tumor size. PATCAM assumes that all PTC cases prior to 1990 were detected through the palpable pathway. Ultrasonography referral rates in the US have substantially increased over time.^21^ To account for this increase, PATCAM incorporates ultrasonography utilization data from Kaiser Permanente Washington starting in 1997 to estimate the yearly probability of referral to ultrasonography in the nonpalpable pathway (eFigure 1 in Supplement 1). For example, the probability of ultrasonography referral for men in 2000 was 40% higher than that in 1997. From 1990 to 1996, a linear extrapolation was used to project the gradual increase in thyroid ultrasonography referral rates.

### Estimating Rates of Overdiagnosis

The model simulates death from causes unrelated to PTC, regardless of the PTC-related outcomes^28^; that is, each patient has a death date from both PTC and unrelated causes. If death from unrelated causes occurs before a PTC-related death, it is considered to be an overdiagnosed case. In the present study, overdiagnosis was defined as the number of persons with PTC who did not die from their cancer (numerator) divided by the total number of persons with PTC (denominator).^8^

To obtain an estimate of the proportion of overdiagnosed cases, we simulated 2 scenarios in which individuals with a SEER historical stage of localized or regional PTC, who would normally be diagnosed and treated immediately, were left untreated. *Localized* refers to cancers confined to the thyroid gland, and *regional* refers to cancer spread to nearby nonvital structures, such as muscle and lymph nodes. In the model, these localized and regional stage cancers are allowed to continue to exist and grow in the simulation according to their individual natural history until they reach a SEER historical distant stage, at which point treatment is started. In clinical practice, it would be unethical to let PTC progress untreated until it is at distant stage, but the model allowed us to test the counterfactual—the population mortality outcome—were this to occur. We estimated the mortality risks if these PTC cases remained untreated under 2 different scenarios.

The first scenario was the lower-bound scenario, which assumes that all tumors instantaneously become a SEER distant stage and start receiving treatment. Namely, all localized and regional PTC cases are assigned PTC-related death dates that align with the SEER distant stage survival rates. This lower-bound scenario imposes the highest mortality rates and thus minimizes the rate of overdiagnosis. Specifically, individuals diagnosed with localized and regional stage PTCs have more indolent cancers and receive treatment, which confers higher survival rates, commensurate with their stage. SEER survival rates are more favorable for localized and regional stage PTCs (10-year survival rates of 99.4% and 96.5%, respectively) than for distant stage PTC (10-year survival rate of 74.6%) (eFigure 2 in Supplement 1).

The second scenario was the upper-bound scenario, which assumes that all localized or regional tumors remain untreated unless they progress to the SEER distant stage according to the natural history of the disease, at which point they are treated per standard of care. This scenario imposes the lowest mortality rates, those commensurate with early-stage PTC, and thus maximizes the rate of overdiagnosis.

### Sensitivity Analysis

The rapid increase in thyroid cancer incidence over the past decades has been attributed to 2 factors: increased detection of clinically inactive cancers, and a true increase from higher biological risk of developing cancer. Either or both of these phenomena may be occurring. To account for the possibility of higher biological risk, our base-case natural history component assumed that the underlying risk of developing thyroid cancer increased 1% annually since 1975 (1.01^35 years^ – 1 = 42% increase by 2010), independent of increased detection.^34^ To examine the implications of this crucial assumption for the rates of overdiagnosis, we performed sensitivity analyses assuming that the risk of thyroid cancer increased 3% annually since 1975 (1.03^35 years^ – 1 = 181% increase by 2010) and, separately, that the risk did not increase at all (eMethods in Supplement 1). For each scenario, we recalibrated PATCAM to align with the incidence rates reported by SEER. These analyses provide insights into how variations in the underlying biological risk might affect thyroid cancer incidence.

### Statistical Analysis

We ran 100 independent replications of PATCAM, with each replication simulating half of the US population. Simulations were conducted under identical conditions to maintain consistency across replications, with each run using the same parameters except for the stochastic elements, including the random seed values. Given the large sample size for the simulations and the stability of the model outcomes, the 95% CIs for the key metrics were notably narrow. Therefore, we report model estimations as the range between the lower- and upper-bound scenarios using only the mean values for clarity. Following the SEER program standard approach for age-adjustment, all outcomes (incidence and mortality) were age-adjusted using the year 2000 US population. Absolute rates are reported per 100 000 individuals.

In a secondary analysis, we evaluated the association of varying ultrasonography referral patterns with incidence and all-cause mortality of PTC over time. Four scenarios were simulated. Scenario 1 used contemporary observed ultrasonography referral patterns using Kaiser Permanente Washington data, whereas the other 3 scenarios incrementally reduced the rates of referral from the observed rate for nonpalpable nodules since 1990. Specifically, scenarios 2, 3, and 4 assumed that ultrasonography referral rates for nonpalpable nodules over time were reduced by 33% (moderate reduction), 67% (substantial reduction), and 100% (complete elimination), respectively. In each of these simulations, we assumed that the process for the detection of the palpable tumors remained the same over time. Data analysis was conducted from June 2024 to August 2025 using Python 3.11 and 3.13 (Python Software Foundation).

## Results

**Figure 1.**
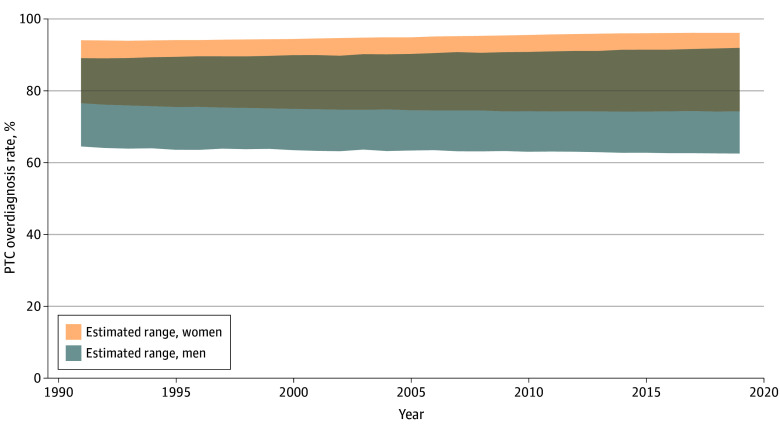
Line Graph of Proportion of Papillary Thyroid Cancer (PTC) Overdiagnosis in the US by Sex From 1991 to 2019

The simulation included all adults, aged 18 years or older, in the US. PATCAM estimated that between 1991 and 2019, 72% to 94% of PTC cases in the US were overdiagnosed, meaning 72% to 94% of PTC cases diagnosed did not affect population mortality. More women were overdiagnosed than men (lower to upper bound range: 75%-95% vs 63%-90%) (Figure 1), and this finding was consistent across nearly all age groups (Table). Over 3 decades, the percentage of individuals overdiagnosed with PTC was relatively stable by age group and sex.

**Table.** Proportion of Overdiagnosed Papillary Thyroid Cancers Among All Diagnosed Cases Stratified by Sex and Age Group, 1991-2019

Group	Lower bound to upper bound range^a^							
	Overall		1991-2000		2001-2010		2011-2019	
	Overdiagnosis rate, %	Absolute rate per 100 000 individuals	Overdiagnosis rate, %	Absolute rate per 100 000 individuals	Overdiagnosis rate, %	Absolute rate per 100 000 individuals	Overdiagnosis rate, %	Absolute rate per 100 000 individuals
Overall	72-94	8-11	73-93	5-6	72-94	8-11	71-95	12-15
Women, all ages	75-95	13-17	76-94	8-10	75-95	13-17	74-96	18-23
Women aged 15-34 y	81-97	8-10	82-97	5-6	81-97	8-10	81-98	12-14
Women aged 35-49 y	83-95	21-24	84-95	13-14	83-95	22-25	82-96	29-34
Women aged 50-64 y	64-94	15-22	65-92	9-12	64-94	16-23	64-96	20-31
Women Ages ≥65 y	50-93	6-11	50-91	3-6	50-93	6-11	50-96	8-16
Men, all ages	63-90	3-5	64-89	2-3	63-90	3-4	63-91	5-7
Men aged 15-34 y	71-94	2-2	72-93	1-1	71-94	2-2	70-94	3-4
Men aged 35-49 y	72-91	4-6	73-90	2-3	72-91	4-5	71-91	7-9
Men aged 50-64 y	58-88	4-6	58-87	2-3	59-88	4-6	58-90	6-10
Men aged ≥65 y	49-90	4-7	49-89	2-4	49-89	4-7	49-91	6-11

^a^ The range includes lower- and upper-bound Papillary Thyroid Carcinoma Microsimulation Model estimates. Lower-bound scenario assumes all tumors instantly become a distant stage in the Surveillance, Epidemiology, and End Results (SEER) program and start receiving treatment, which imposes the highest mortality rate and thus minimizes the rate of overdiagnosis. Upper-bound scenario assumes all localized or regional tumors remain untreated unless they progress to the SEER distant stage according to the natural history of the disease, which imposes the lowest mortality rate and thus maximizes the rate of overdiagnosis.

### Absolute Rates of Overdiagnosis

From 1991 to 2019, 443 212 to 573 705 women and 107 804 to 154 504 men were overdiagnosed with PTC. Over this period, women had a higher absolute rate of overdiagnosis than men (13-17 per 100 000 individuals vs 3-5 per 100 000 individuals), with the greatest number of cases observed among women aged 35 to 49 years (21-24 per 100 000 individuals) and 50 to 64 years (15-22 per 100 000 individuals) (Table). Overdiagnosis rates in women were lowest in the youngest age group (15-34 years of age). However, even in this group, women had a higher rate of overdiagnosis than men (8-10 per 100 000 individuals vs 2-2 per 100 000 individuals).

**Figure 2.**
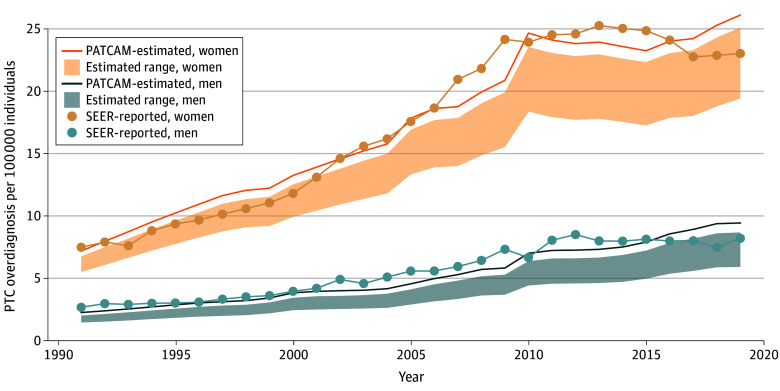
Line Graph of Absolute Rates of Papillary Thyroid Cancer (PTC) Overdiagnosis in the US Stratified by Sex From 1991 to 2019 The range includes lower- and upper-bound estimates from the Papillary Thyroid Carcinoma Microsimulation Model (PATCAM). Lower-bound scenario assumes all tumors instantly become a distant stage in the Surveillance, Epidemiology, and End Results (SEER) program and start receiving treatment. Upper-bound scenario assumes all localized or regional tumors remain untreated unless they progress to the SEER distant stage according to the natural history of the disease.

Absolute rates of overdiagnosis substantially increased between 1991 and 2019 for both sexes (Figure 2) and across all age groups (Table). Over this period, overdiagnosis increased for both women (8-10 per 100 000 individuals [1991-2000] to 18-23 per 100 000 individuals [2011-2019]) and men (2-3 per 100 000 individuals [1991-2000] to 5-7 per 100 000 individuals [2011-2019]). The greatest increases were observed among women aged 35 to 49 years (13-14 per 100 000 individuals [1991-2000] to 29-34 per 100 000 individuals [2011-2019]) and aged 50 to 64 years (9-12 per 100 000 individuals [1991-2000] to 20-31 per 100 000 individuals [2011-2019]).

### Implications of Changing Biological Risk Assumptions

The model reproduced observed SEER incidence and mortality over time after changing the baseline 1% annual increased biological risk of developing PTC down to 0% and up to 3% (eFigures 3-5 in Supplement 1). Even in an extreme situation where the underlying risk of thyroid cancer was increased by 3% per year, representing a 181% increase in underlying risk since 1975, our model showed that the contribution of a true increase in biological risk to the increase in thyroid cancer incidence remained negligible (Table; eTables 2-3 and eFigures 3-5 in Supplement 1).

### Implications of Reduced Ultrasonography Referral for Incidence and Mortality

**Figure 3.**
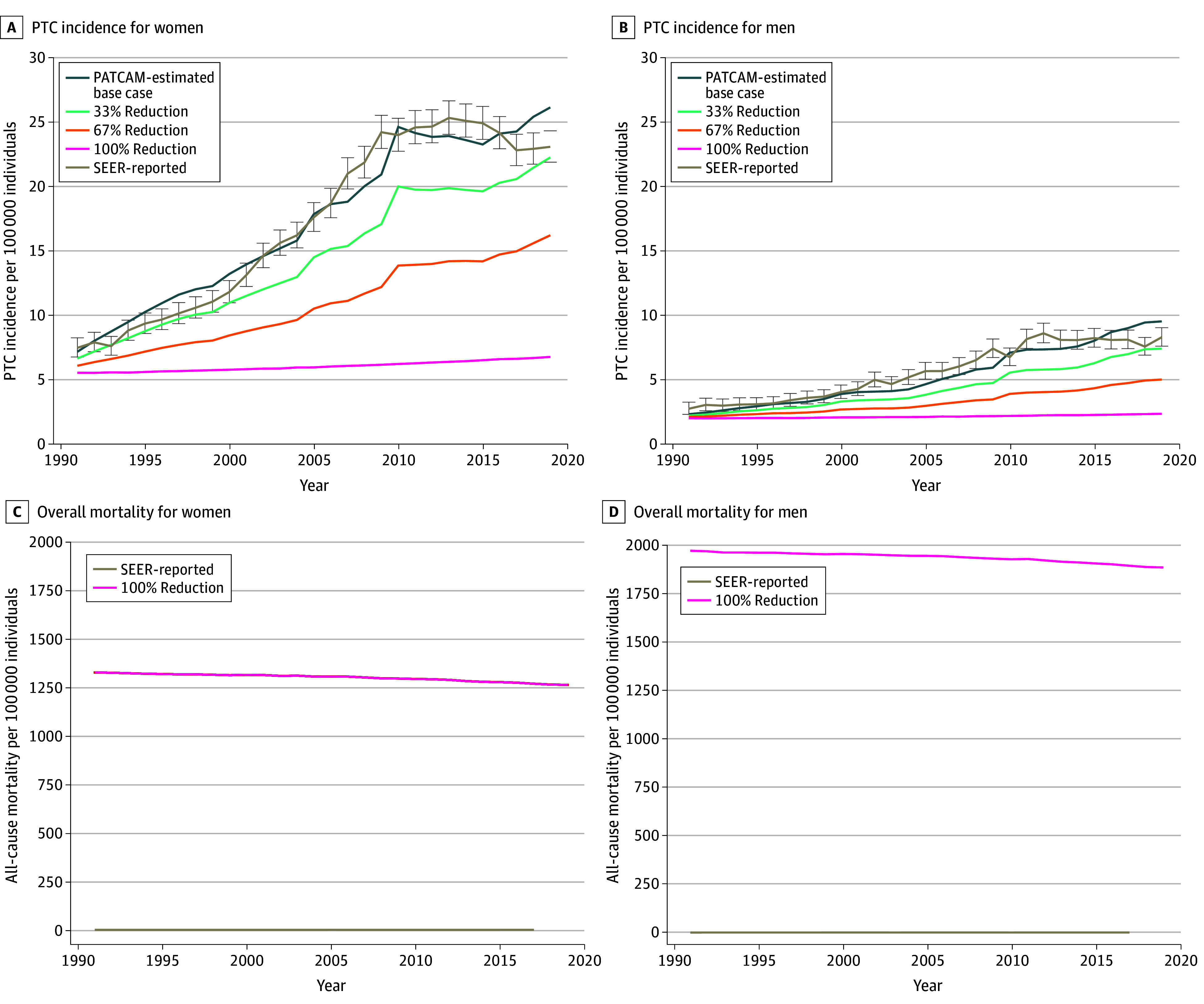
Line Graphs of Association of Reducing Thyroid Ultrasonography Referral Rates With Papillary Thyroid Cancer (PTC) Incidence and Overall Mortality in the US Stratified by Sex From 1991 to 2019 The Surveillance, Epidemiology, and End Results (SEER) program–reported value is less than 1 mortality per 100 000 individuals, while the base case, 33% reduction, 67% reduction, and 100% reduction scenarios are nearly identical and overlap, almost lying on the same line. PATCAM indicates Papillary Thyroid Carcinoma Microsimulation Model.

PATCAM estimated that reducing ultrasonography referral for nonpalpable nodules by 33% and 67% would have led to a 17% decrease (18 to 15 per 100 000 individuals) and 41% decrease (18 to 11 per 100 000 individuals) in PTC incidence in 2019, respectively, regardless of sex, with the greatest decrease in absolute rates occurring among women (Figure 3). These reductions resulted in a less than 0.1% change in overall mortality between 1991 and 2019 (Figure 3).

Eliminating the use of ultrasonography (100% referral reduction) for nonpalpable thyroid nodules would have resulted in a dramatic decrease in the PTC incidence rate in 2019, bringing it down to 4.5 per 100 000 individuals, on par with the rate observed in SEER in 1991, when the rate was 3.8 per 100 000 individuals, with a similar negligible change in overall mortality (<0.1%) (Figure 3). However, when ultrasonography was not used for nonpalpable indications, PATCAM estimated there would be a slow, steady increase in the incidence of palpable cancers, with an annual increase of 0.6% for women and 0.5% for men between 1991 and 2019. Age group–specific results estimated that reduction in the use of ultrasonography for nonpalpable nodules would have resulted in the greatest decrease in absolute PTC incidence rates among women aged 35 to 64 years (eFigure 6 in Supplement 1). Additional findings are available in eFigure 7 in Supplement 1.

## Discussion

Using PATCAM, a validated population model of thyroid cancer epidemiological patterns,^28^ we estimated that at least 72% and as much as 94% of PTC cases diagnosed in the US between 1991 and 2019 were overdiagnosed. Identification of those cancers and the treatment of those patients did not benefit population mortality. However, it resulted in hundreds of thousands of people living with the psychological burden of a cancer diagnosis and the adverse effects of treatment. Even accounting for a true underlying increase in the risk of developing thyroid cancer over this time frame,^36^ our estimates confirmed that the detection of clinically inactive cancers is the primary factor in the rapid increase in thyroid cancer over the past 3 decades. Women had overdiagnosis rates that were 3 to 4 times higher than for men, with an estimated 443 212 to 573 705 women affected compared with 107 804 to 154 504 men. The greatest absolute rate increases were among women aged 35 to 64 years.

Our finding that an estimated 72% to 94% of PTC cases were overdiagnosed is notably higher than rates previously reported,^37,38^ primarily because of differences in methods. We used a microsimulation model, while other investigators compared the ratio of observed-to-expected thyroid incidence.^38,39^ The observed-to-expected method has several limitations, which are overcome by simulation modeling. First, prior estimates did not account for other-cause mortality. In the observed-to-expected method, individuals 65 years or older were the referent category (expected rate denominator); thus, it was assumed that older individuals are not overdiagnosed. This assumption is problematic because older adults are often overdiagnosed. Second, prior estimates did not incorporate the natural history of thyroid cancer in their models, instead focusing on historical cross-sectional age-specific incidence rates to make calculations. Third, prior estimates did not explicitly consider the role of changes in the underlying risk of thyroid cancer over time, as our model did. Instead, prior approaches used pre-1975 thyroid cancer incidence curves from Nordic countries that were collected prior to diagnostic changes.

We acknowledge that the concept of overdiagnosis, which is about population mortality benefit, does not account for the need for clinicians in practice to tolerate some degree of overdiagnosis. For any given patient, the ultimate clinical trajectory cannot be known in advance, and thyroid cancers can substantially affect quality of life if allowed to grow unchecked. Nevertheless, the rate of overdiagnosis estimated by our model was high enough to suggest that many more patients are receiving thyroid cancer diagnoses and undergoing surgeries than is necessary considering the potential harms of diagnosis and surgery relative to the oncologic benefit.^40^

The diagnostic pathway to thyroid cancer starts with ultrasonography referral. Nevertheless, little has been done to guide referral decisions. There is a low threshold for referral for potentially thyroid-attributable signs and symptoms (eg, hoarseness, globus), as ultrasonography has the advantage of easy access compared with other imaging modalities, portability, and no radiation exposure. Contemporary multi-institutional studies have shown that approximately 80% of thyroid ultrasonography referrals are for evaluation of vague symptoms such as globus pharyngeus, are ordered as part of broad investigations into constitutional symptoms (eg, fatigue), or requested due to patient or clinician concern for an uncertain physical finding.^41^ Ultrasonography for these indications often results in nodule detection, which rarely explains the symptoms or physical examination concern of the patient.^41^ While ultrasonography is low risk, thyroid nodules are extremely common, and their detection—regardless of whether ultrasonography answered the question being asked on the referral—sets off a cascade of decision-making about biopsy and management. For these reasons, providing guidance for when to order an ultrasonography, particularly for nonpalpable, asymptomatic thyroid nodules, is an important target for future interventions to reduce thyroid cancer overdiagnosis.

Our findings support efforts to reduce ultrasonography referrals. A 33% and 67% simulated reduction in ultrasonography for nonpalpable nodules reduced PTC incidence by 17% and 41%, respectively. Complete elimination of ultrasonography use for nonpalpable indications reduced thyroid cancer incidence to levels observed in 1991. In all of these simulations, there was negligible implication for population mortality (Figure 3). Our model did estimate that, with complete elimination of ultrasonography for nonpalpable nodules, there would be a slow but steady increase in the incidence of palpable cancers, with an annual increase of 0.6% for women and 0.5% for men between 1991 and 2019. This estimate indicates that an ultrasonography guideline reducing PTC incidence by even 17% would result in only minimal increases in the proportion of palpable nodules—lesions that remain readily treatable using the same approaches applied to smaller, screen-detected nodules and cancers.

Efforts to reduce ultrasonography for nonpalpable nodules would benefit women the most. Data show that women have a higher overdiagnosis rate than men primarily because they are referred for ultrasonography at a higher rate.^42^ While there have been advances in recent years in thyroid cancer treatment deescalation, from total thyroidectomy with radioactive iodine for almost all patients to thyroid lobectomy or even active surveillance for the smallest cancers, active surveillance is offered in only a few places in the US.^43,44^ As a treatment, active surveillance effectively limits treatment-related adverse effects, but it does require that patients interact with the health care system, with incumbent loss of time and costs of care. These burdens would not be incurred in the counterfactual scenario where their small, indolent cancer was never detected. Current standards still dictate that the vast majority of patients undergo total thyroidectomy or thyroid lobectomy, resulting in quality-of-life consequences related to treatment adverse effects as well as psychological adverse effects related to living with a cancer diagnosis.

This study simulated counterfactual population-level scenarios, not individual clinical decisions; therefore, its conclusions pertain to population-level overdiagnosis and should not be interpreted as guidance for the management of a specific thyroid nodule or patient. Currently, the model cannot determine which nodules should be detected. This feature is the focus of future work that will integrate risk stratification, sonographic features, and treatment benefit-harm tradeoff into the model to better inform recommendations about which patients may experience net benefit from detection and intervention.

### Limitations

Our study has limitations. We focused solely on the implications of PTC for population mortality and did not evaluate the potential quality-of-life consequences of thyroid cancer for swallowing, speaking, and breathing through local growth. Nevertheless, the results suggest considerable opportunity to deescalate identification and treatment of PTCs without increasing population mortality. The model was developed and validated using high-quality data that were carefully vetted prior to inclusion and had high accuracy in estimating rates of tumors.^26,34,35^ It is possible that not all data are generalizable, such as if ultrasonography and indication data from Kaiser Permanente Washington are different from those in clinical practice elsewhere. However, Kaiser Permanente data have been shown to be generalizable to the US population,^45,46,47,48^ and Kaiser Permanente cancer data mirror those of the Puget Sound SEER registry, which has thyroid cancer incidence rates that are similar to those of the overall US population. Using these data, PATCAM was able to replicate US cancer incidence rates as reflected in SEER. We performed careful sensitivity analyses to evaluate the robustness of our estimates and externally validated the model to evaluate representativeness of these data.

## Conclusions

In this study, we estimated that between 72% and 94% of PTC cases diagnosed in the US between 1991 and 2019 were overdiagnosed, even after accounting for an underlying increased risk in the development of thyroid cancer. Our findings suggest that the detection of these cancers and their treatment yielded no benefit to population mortality. Although some degree of overdiagnosis is unavoidable in clinical practice, the persistently high rates observed suggest an opportunity to reduce unnecessary thyroid ultrasonography referrals, particularly for nonpalpable nodules. Doing so could lower the number of cancer diagnoses and associated psychological and treatment burdens without compromising population health or increasing mortality.

## Notes

Supplement 1. **eTable 1.** Data Sources Used to Inform the PATCAM Model **eFigure 1**. Annual Adjustment Factors Used in PATCAM **eFigure 2.** Stage-Specific Survival Based on Reported SEER Rates
eMethods.
**eTable 2.** Proportion of Overdiagnosis by Sex, Age Group, and Year When the Annual Risk Increase Is Equal to 0% **eFigure 3.** Overdiagnosis Estimates by Sex and Age Group Over Time When Annual Risk Increase Is Equal to 0% **eFigure 4.** Overdiagnosis Estimates by Sex and Age Group Over Time When Annual Risk Increase Is Equal to 1% **eTable 3.** Proportion of Overdiagnosis by Sex, Age Group, and Year When the Annual Risk Increase Is Equal to 3% **eFigure 5.** Overdiagnosis Estimates by Sex and Age Group Over Time When Annual Risk Increase Is Equal to 3% **eFigure 6.** Impact of Reduction in Thyroid Ultrasound Referral Rates on Thyroid Cancer Incidence **eFigure 7.** Impact of Reduction in Thyroid Ultrasound Referral Rates on Thyroid Cancer Mortality
eReferences

Supplement 2.
Data Sharing Statement
