# Error in the Byline

**DOI:** 10.1001/jamanetworkopen.2026.9142

**Published:** 2026-04-01

In the Original Investigation titled “Overdiagnosis of Papillary Thyroid Cancer,”^1^ published on February 24, 2026, there was an error in the byline. Dr Cher’s name should appear as follows: Benjamin A. Y. Cher, MD. The article has been corrected.
