# Supplementary material for: Overdiagnosis of Papillary Thyroid Cancer
Source: JAMA Netw Open. 2026 Feb 24;9(2):e2559852. doi: 10.1001/jamanetworkopen.2025.59852 (PMC12933285; doi:10.1001/jamanetworkopen.2025.59852)
Supplement: Supplement 1. — eTable 1. Data Sources Used to Inform the PATCAM Model eFigure 1. Annual Adjustment Factors Used in PATCAM eFigure 2. Stage-Specific Survival Based on Reported SEER Rates eMethods. eTable 2. Proportion of Overdiagnosis by Sex, Age Group, and Year When the Annual Risk Increase Is Equal to 0% eFigure 3. Overdiagnosis Estimates by Sex and Age Group Over Time When Annual Risk Increase Is Equal to 0% eFigure 4. Overdiagnosis Estimates by Sex and Age Group Over Time When Annual Risk Increase Is Equal to 1% eTable 3. Proportion of Overdiagnosis by Sex, Age Group, and Year When the Annual Risk Increase Is Equal to 3% eFigure 5. Overdiagnosis Estimates by Sex and Age Group Over Time When Annual Risk Increase Is Equal to 3% eFigure 6. Impact of Reduction in Thyroid Ultrasound Referral Rates on Thyroid Cancer Incidence eFigure 7. Impact of Reduction in Thyroid Ultrasound Referral Rates on Thyroid Cancer Mortality eReferences [file jamanetwopen-e2559852-s001.pdf]

Supplemental Online Content

Francis DO, Davies L, Zhang Y, et al. Overdiagnosis of papillary thyroid cancer. *JAMA Netw Open*. 2026;9(2):e2559852.
doi:10.1001/jamanetworkopen.2025.59852

eTable 1. Data Sources Used to Inform the PATCAM Model

eFigure 1. Annual Adjustment Factors Used in PATCAM

eFigure 2. Stage-Specific Survival Based on Reported SEER Rates

eMethods.

eTable 2. Proportion of Overdiagnosis by Sex, Age Group, and Year When the Annual Risk Increase Is Equal to 0%

eFigure 3. Overdiagnosis Estimates by Sex and Age Group Over Time When Annual Risk Increase Is Equal to 0%

eFigure 4. Overdiagnosis Estimates by Sex and Age Group Over Time When Annual Risk Increase Is Equal to 1%

eTable 3. Proportion of Overdiagnosis by Sex, Age Group, and Year When the Annual Risk Increase Is Equal to 3%

eFigure 5. Overdiagnosis Estimates by Sex and Age Group Over Time When Annual Risk Increase Is Equal to 3%

eFigure 6. Impact of Reduction in Thyroid Ultrasound Referral Rates on Thyroid Cancer Incidence

eFigure 7. Impact of Reduction in Thyroid Ultrasound Referral Rates on Thyroid Cancer Mortality

eReferences

This supplemental material has been provided by the authors to give readers additional information about their work.

© 2026 Francis DO et al. *JAMA Network Open*

Appendix A. Supporting Data and Adjustment Methodology for PATCAM

eTable 1. Data sources used to inform the PATCAM model

	Name	Dependence	Data source
Population demographics	Population over time	Age and sex	US Census Data[1]
	Other-cause mortality	Age and sex	Human Mortality Database[2]
Natural history	Relative distribution of initiating thyroid nodules	Age and sex	Reiners, et al[3]*
Tumor detection	Probability of thyroid ultrasound-palpable	Tumor size, sex, age group	KPWA** data and calibrated
	Probability of thyroid ultrasound-non-palpable	Tumor size, sex, age group, year	KPWA data and calibrated
	Probability of biopsy referral after ultrasound	Tumor size	KPWA data and calibrated
	Sensitivity of biopsy	None	Systematic Review and Meta-Analysis[4,5]
Post-diagnosis	Survival after diagnosis	Age, sex, tumor stage	SEER[6]***

* Cross-sectional study on thyroid size and nodule initiation; largest study of this type in the literature

** KPWA = Kaiser Permanente Washington

***SEER = Surveillance, Epidemiology, and End Results Program

© 2026 Francis DO et al. *JAMA Network Open*

eFigure 1. Annual adjustment factors used in PATCAM

To account for temporal changes in clinical practice patterns, we developed an **annual adjustment factor** that modifies the baseline probability of referral to thyroid ultrasound for nonpalpable papillary thyroid carcinomas (PTCs) in the PATCAM model. This factor is derived from KPWA data and reflects the evolving likelihood of referral over time, capturing both observed trends and necessary adjustments to align with broader epidemiological patterns. Between 1993 and 1996, we smoothed the KPWA referral data to mitigate abrupt variations and assumed a linear increase in referral rates from 0% in 1990 to the observed levels by 1996. Additionally, due to limited sample sizes, we combined referral data across four age groups (15–34, 45–49, 50–64, and 65+). To better match SEER incidence trends, we reduced referral probabilities by 10% before 2000 and increased them by 10% after 2005. The resulting annual adjustment factor is applied each year in PATCAM to scale the probability of referral.

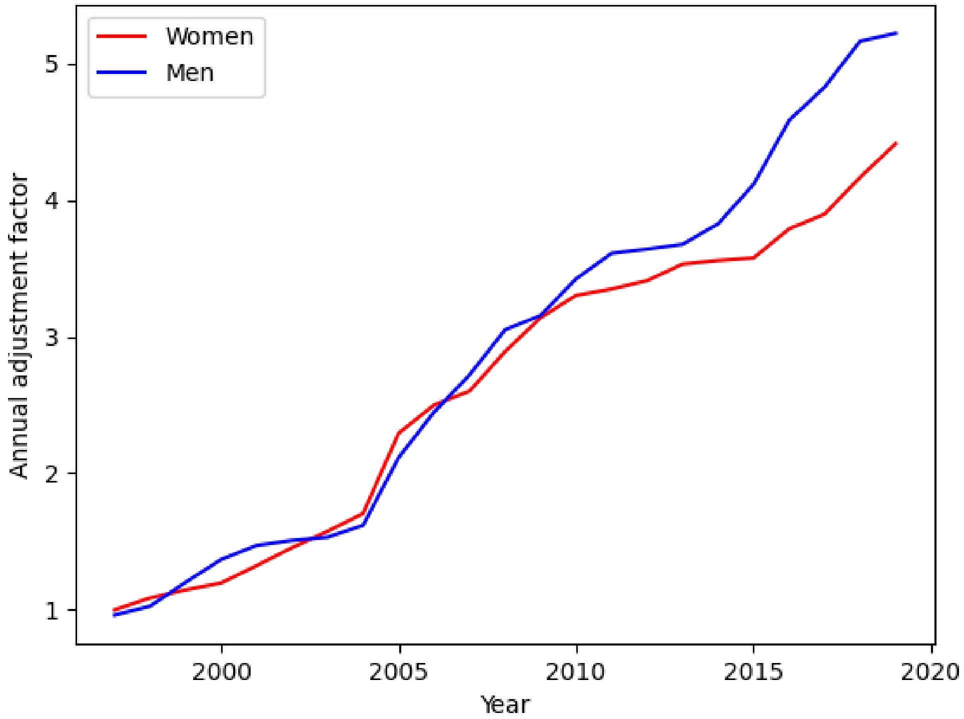

© 2026 Francis DO et al. *JAMA Network Open*

Appendix B. Stage-specific survival based on reported SEER rates

eFigure 2. Stage-specific survival based on reported SEER rates.

A) Stage-specific survival probability for women between ages 15-34; B) Stage-specific survival probability for women between ages 35-49; C) Stage-specific survival probability for women between ages 50-64; D) Stage-specific survival probability for women aged over 65; E) Stage-specific survival probability for men between ages 15-34; F) Stage-specific survival probability for men between ages 35-49; G) Stage-specific survival probability for men between ages 50-64; H) Stage-specific survival probability for men aged over 65.

A) Women ages 15-34y

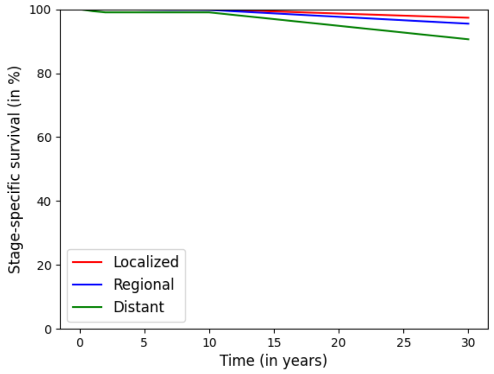

B) Women ages 35-49y

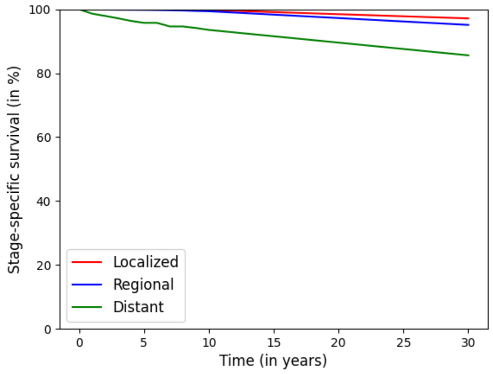

C) Women ages 50-64y

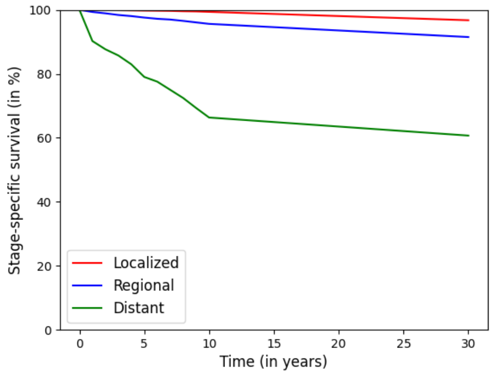

D) Women ages 65+y

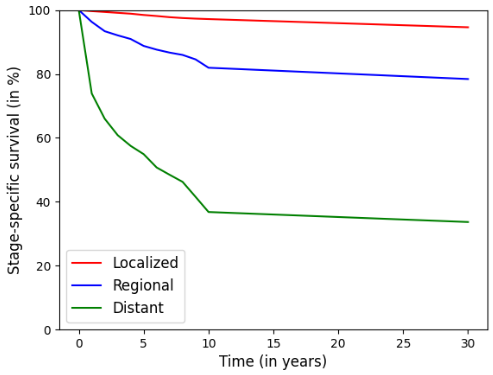

© 2026 Francis DO et al. *JAMA Network Open*

E) Men ages 15-34y

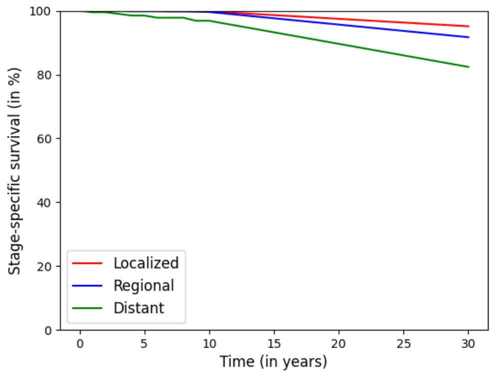

F) Men ages 35-49y

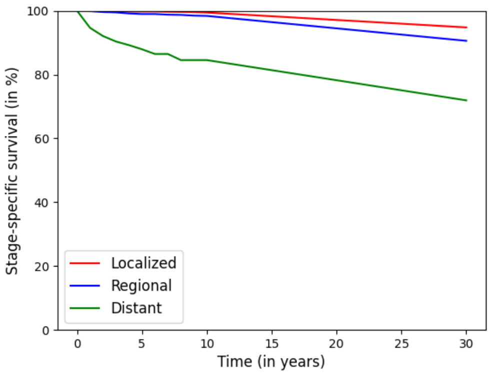

G) Men ages 50-64y

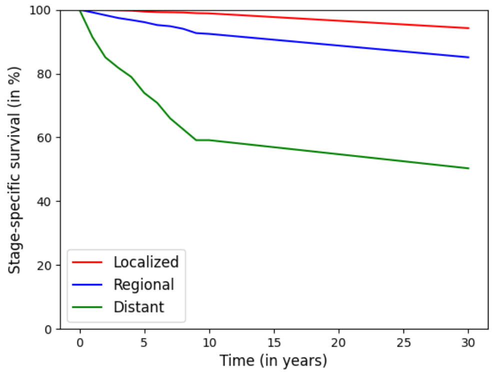

H) Men ages 65+yG)

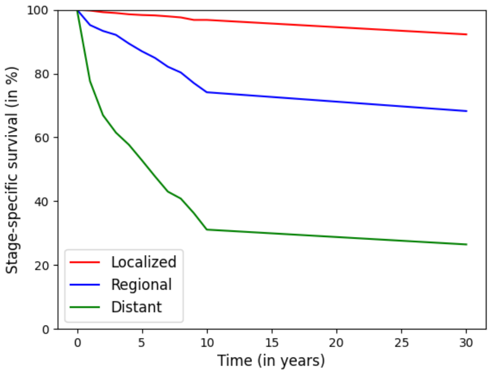

© 2026 Francis DO et al. *JAMA Network Open*

Appendix C. Details of the sensitivity analysis results

eMethods

As noted in the main text, the PATCAM model accounts for the increased biological risk of developing thyroid cancer by incorporating the *annual risk increase* parameter, defined as the annual percentage increase in the risk of developing thyroid cancer since 1975. We conducted a sensitivity analysis by adjusting this parameter from PATCAM's baseline of 1% to evaluate the relative impact of overdetection versus an actual increase in biological risk on thyroid cancer incidence rates.

Sections C.1-C.3 present the results when the annual risk increase is set to 0%, 1%, and 3%, respectively. **eTables 2-3** show the proportion of overdiagnosed cases and absolute rates of overdiagnosis per 100,000 individuals by sex, age group, and three periods (1991-2000, 2001-2010, and 2011-2019) at 0% and 3%. These values are derived by calculating the average of the rates of overdiagnosis included in each time period. **eFigure 3-5** show the proportion of overdiagnosed cases and absolute rates of overdiagnosis per 100,000 individuals over time by sex and age group and 0%, 1%, and 3%.

C.1. Model projections when the annual risk increase is equal to 0%

eTable 2. Proportion of overdiagnosis by sex, age group, and year when the annual risk increase is equal to 0%.
Numbers in parentheses represent the absolute rates of overdiagnosis per 100,000 individuals.

Sex-age group combination	Overall	1991-2000	2001-2010	2011-2019
Overall	72%-94% (7-10)	73%-93% (5-6)	72%-94% (8-10)	71%-95% (10-13)
Women All age	75%-95% (11-14)	76%-94% (7-9)	74%-95% (12-15)	74%-96% (15-19)
Women Age 15-34	82%-97% (7-8)	82%-97% (5-6)	82%-97% (7-8)	81%-98% (9-11)
Women Age 35-49	83%-95% (18-21)	84%-95% (12-14)	83%-95% (20-23)	83%-95% (24-28)
Women Age 50-64	64%-94% (13-19)	64%-92% (8-12)	64%-94% (14-21)	64%-96% (17-26)
Women Age 65+	50%-93% (6-11)	50%-91% (3-6)	50%-93% (6-11)	50%-96% (8-16)
Men All age	63%-90% (3-4)	64%-89% (2-3)	63%-90% (3-4)	62%-91% (5-7)
Men Age 15-34	71%-93% (1-2)	73%-93% (1-1)	71%-94% (1-2)	70%-94% (2-3)
Men Age 35-49	72%-91% (4-5)	73%-90% (2-3)	72%-91% (4-5)	71%-91% (6-8)
Men Age 50-64	58%-89% (4-6)	58%-87% (2-4)	59%-89% (4-6)	58%-90% (6-10)

Men Age 65+	50%-90% (4-7)	50%-88% (2-4)	50%-89% (4-7)	50%-91% (6-11)

eFigure 3. Overdiagnosis estimates by sex and age group over time when annual risk increase is equal to 0%.

A) Proportion of overdiagnosed PTCs among women according to lower-bound scenario B) Proportion of overdiagnosed PTCs among men according to lower-bound scenario; C) Proportion of overdiagnosed PTCs among women according to upper-bound scenario D) Proportion of overdiagnosed PTCs among men according to upper-bound scenario; E) Absolute rate of overdiagnosed PTCs among women according to lower-bound scenario F) Absolute rate of overdiagnosed PTCs among men according to lower-bound scenario; G) Absolute rate of overdiagnosed PTCs among women according to upper-bound scenario H) Absolute rate of overdiagnosed PTCs among men according to upper-bound scenario.

A) Women: % Overdiagnosis lower-bound

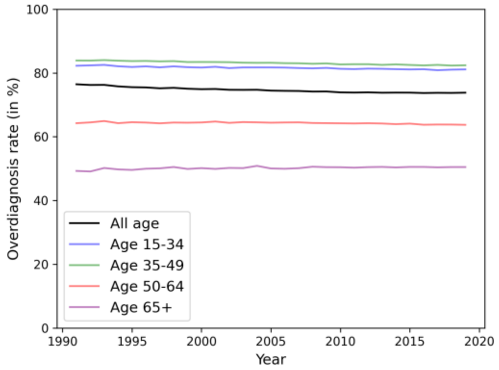

B) Men: % Overdiagnosis lower bound

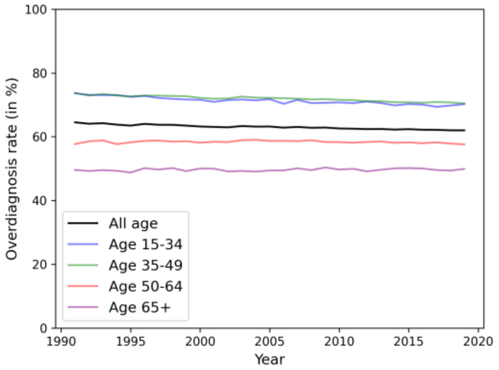

C) Women: % Overdiagnosis upper bound

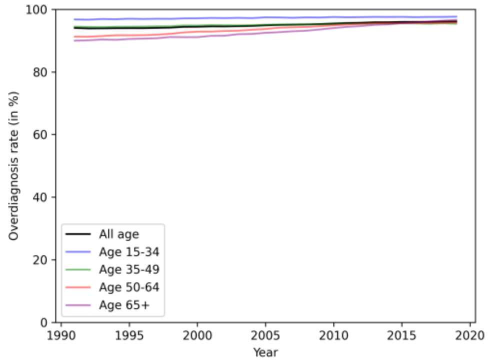

D) Men: % Overdiagnosis upper bound

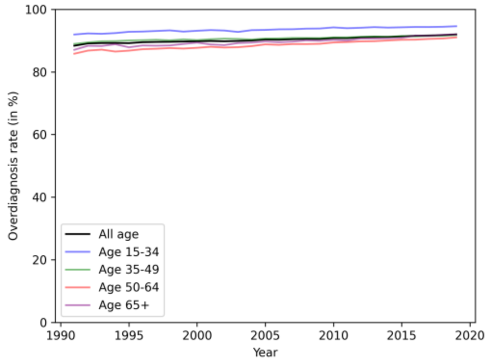

© 2026 Francis DO et al. *JAMA Network Open*

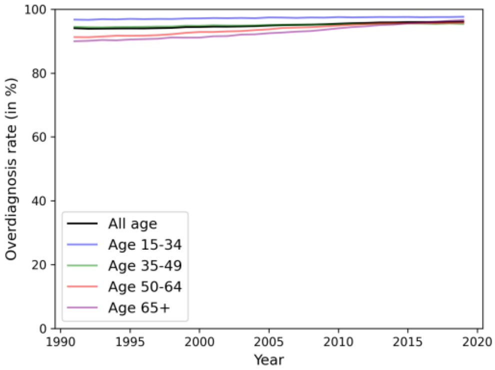

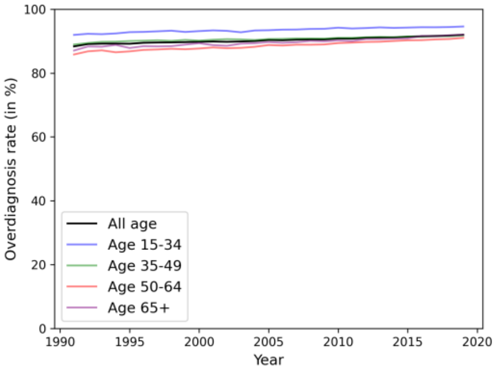

E) Women: Absolute rate lower-bound

F) Men: Absolute rate lower-bound

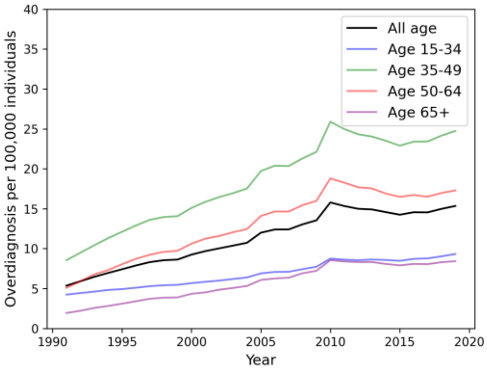

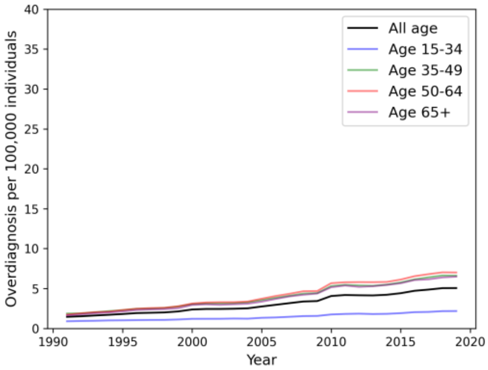

G) Women: Absolute rate upper-bound

H) Men: Absolute rate lower-bound

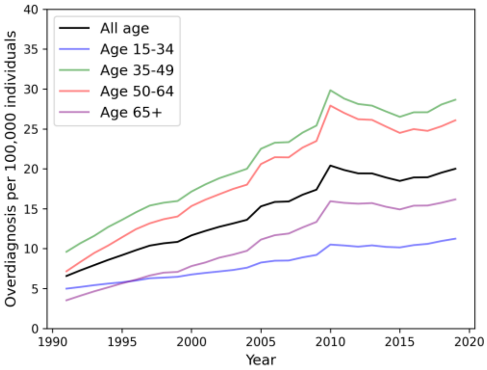

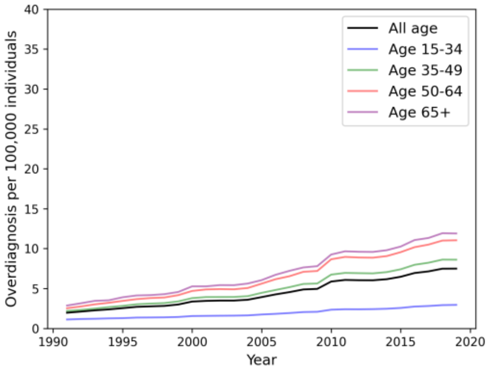

© 2026 Francis DO et al. *JAMA Network Open*

C.2. Model projections when the annual risk increase is equal to 1%

eFigure 4. Overdiagnosis estimates by sex and age group over time when the annual risk increase is equal to 1%.

A) Proportion of overdiagnosed PTCs among women according to lower-bound scenario B) Proportion of overdiagnosed PTCs among men according to lower-bound scenario; C) Proportion of overdiagnosed PTCs among women according to upper-bound scenario D) Proportion of overdiagnosed PTCs among men according to upper-bound scenario; E) Absolute rate of overdiagnosed PTCs among women according to lower-bound scenario F) Absolute rate of overdiagnosed PTCs among men according to lower-bound scenario; G) Absolute rate of overdiagnosed PTCs among women according to upper-bound scenario H) Absolute rate of overdiagnosed PTCs among men according to upper-bound scenario.

A)

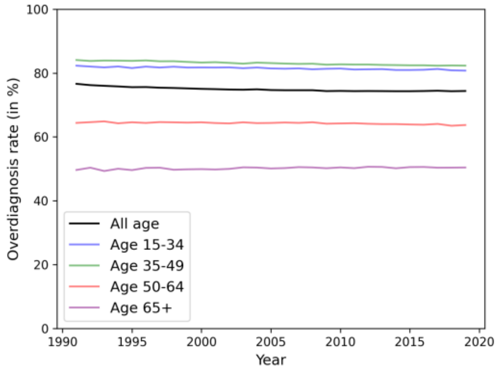

B)

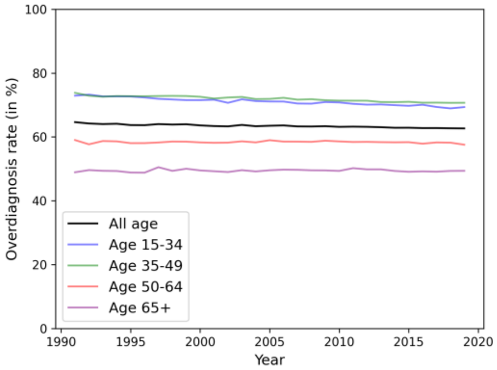

C)

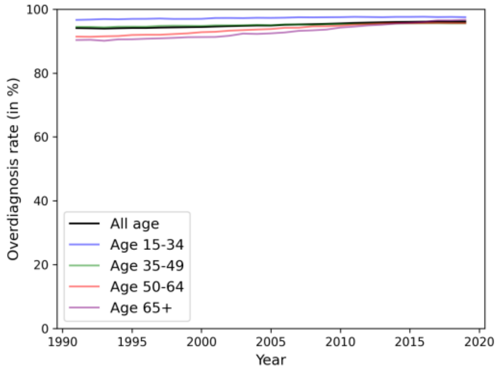

D)

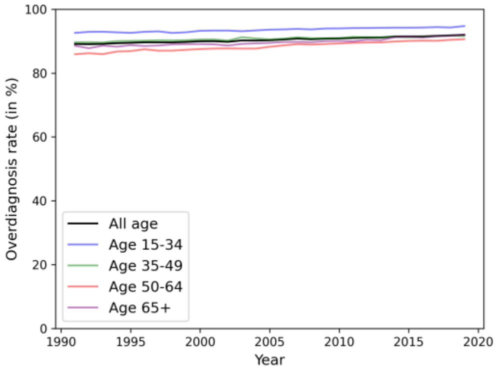

E)

F)

© 2026 Francis DO et al. *JAMA Network Open*

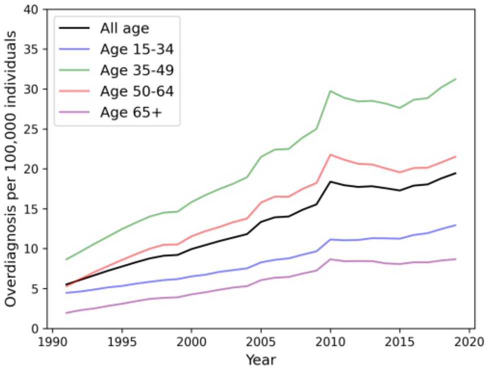

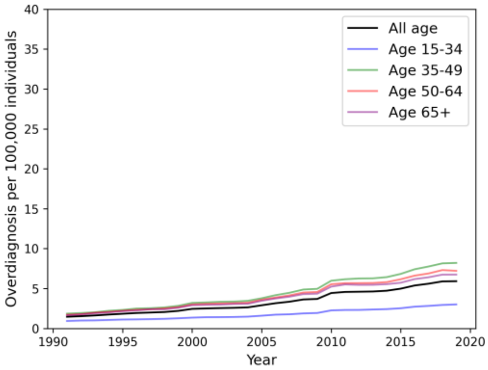

G)

H)

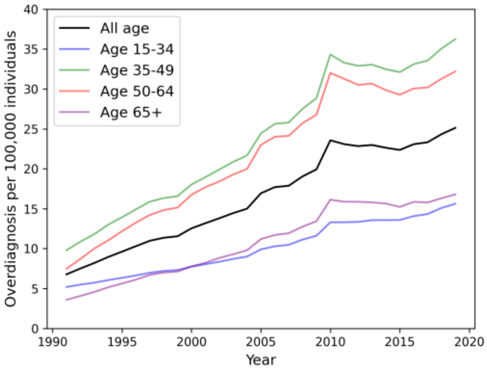

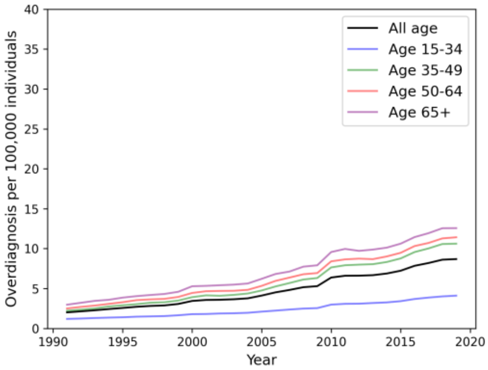

© 2026 Francis DO et al. *JAMA Network Open*

C.3. Model projections when the annual risk increase is equal to 3%

eTable 3. Proportion of overdiagnosis by sex, age group, and year when the annual risk increase is equal to 3%.
Numbers in parentheses represent the absolute rates of overdiagnosis per 100,000 individuals.

**Sex-age group combination**	**1991-2019**	**1991-2000**	**2001-2010**	**2011-2019**
Overall	73%-94% (9-12)	73%-93% (5-6)	72%-94% (9-11)	72%-95% (15-19)
Women All age	76%-95% (14-18)	76%-94% (7-9)	75%-95% (14-18)	75%-96% (23-29)
Women Age 15-34	81%-97% (12-14)	82%-97% (6-7)	81%-97% (11-13)	81%-98% (18-22)
Women Age 35-49	83%-95% (22-26)	84%-95% (11-13)	83%-95% (22-25)	82%-95% (36-42)
Women Age 50-64	64%-93% (13-19)	64%-92% (7-10)	64%-93% (13-19)	64%-94% (20-30)
Women Age 65+	50%-93% (6-12)	50%-91% (3-6)	50%-92% (6-12)	50%-95% (10-19)
Men All age	64%-90% (4-6)	64%-89% (2-3)	64%-90% (4-5)	63%-92% (7-10)
Men Age 15-34	71%-93% (2-3)	72%-93% (1-2)	71%-93% (2-3)	70%-94% (4-5)
Men Age 35-49	72%-91% (5-7)	73%-90% (2-3)	72%-91% (5-6)	71%-91% (9-12)
Men Age 50-64	58%-88% (5-7)	58%-87% (2-3)	58%-88% (4-6)	58%-90% (8-12)
Men Age 65+	49%-90% (4-7)	49%-88% (2-4)	49%-89% (4-7)	49%-91% (6-12)

eFigure 5. Overdiagnosis estimates by sex and age group over time when annual risk increase is equal to 3%.

A) Proportion of overdiagnosed PTCs among women according to lower-bound scenario B) Proportion of overdiagnosed PTCs among men according to lower-bound scenario; C) Proportion of overdiagnosed PTCs among women according to upper-bound scenario D) Proportion of overdiagnosed PTCs among men according to upper-bound scenario; E) Absolute rate of overdiagnosed PTCs among women according to lower-bound scenario F) Absolute rate of overdiagnosed PTCs among men according to lower-bound scenario; G) Absolute rate of overdiagnosed PTCs among women according to upper-bound scenario H) Absolute rate of overdiagnosed PTCs among men according to upper-bound scenario.

A)

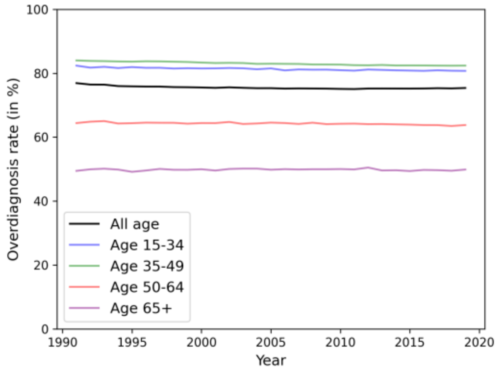

B)

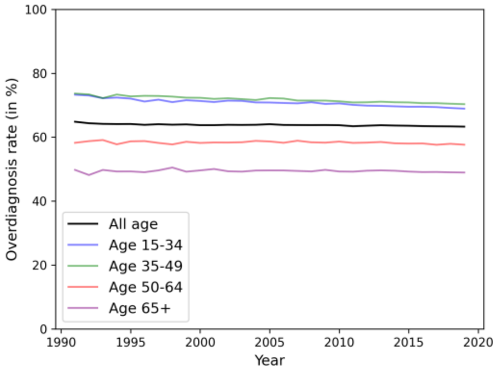

C)

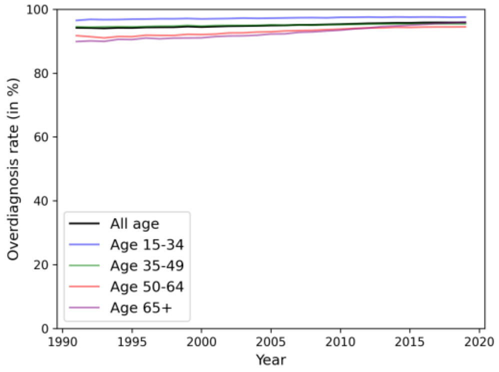

D)

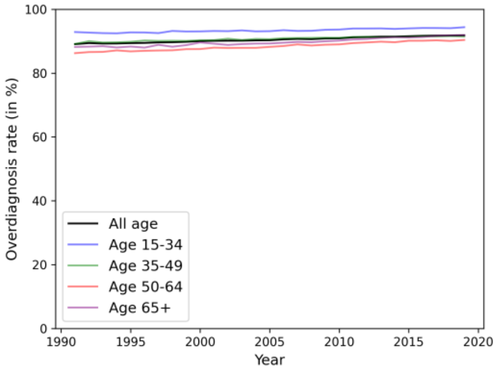

E)

F)

© 2026 Francis DO et al. *JAMA Network Open*

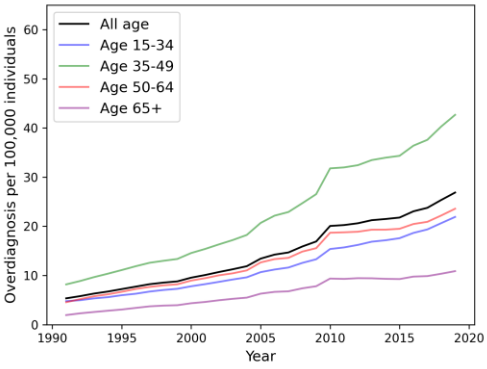

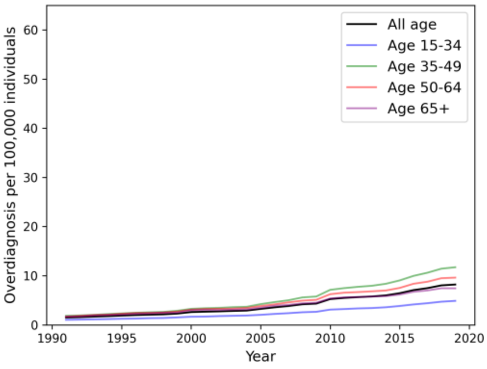

G)

H)

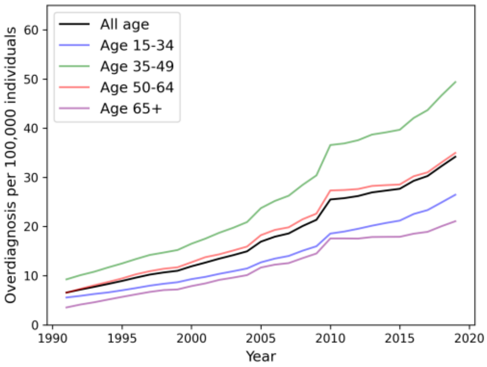

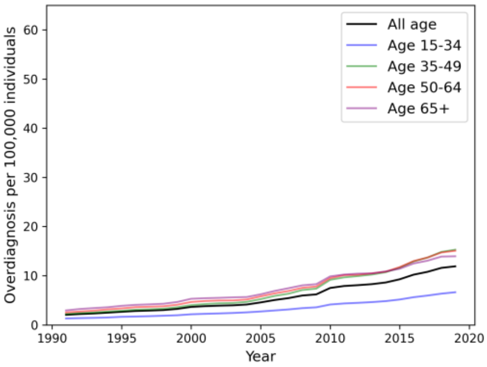

© 2026 Francis DO et al. *JAMA Network Open*

Appendix D. Impact of reduction in thyroid ultrasound referral rates on thyroid cancer incidence and mortality by sex and age group

The following figure shows the results of the secondary analysis by sex and age group.

eFigure 6. Impact of reduction in thyroid ultrasound referral rates on thyroid cancer incidence.
A) Incidence for women aged 15-34; B) Incidence for women aged 35-49; C) Incidence for women aged 50-64; D) Incidence for women aged 65+; E) Incidence for men aged 15-34; F) Incidence for men aged 35-49; G) Incidence for men aged 50-64; H) Incidence for men aged 65+.

A)

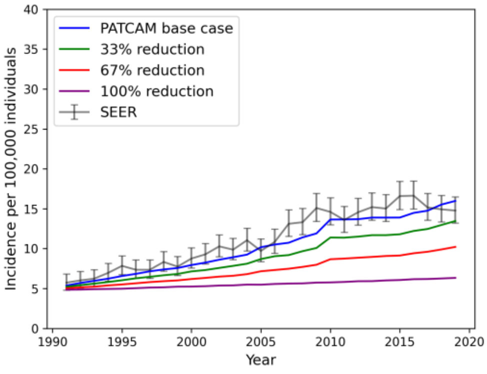

B)

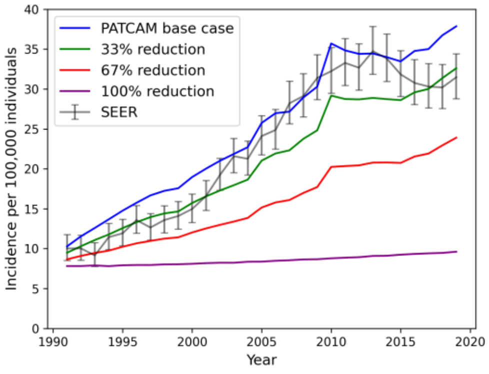

C)

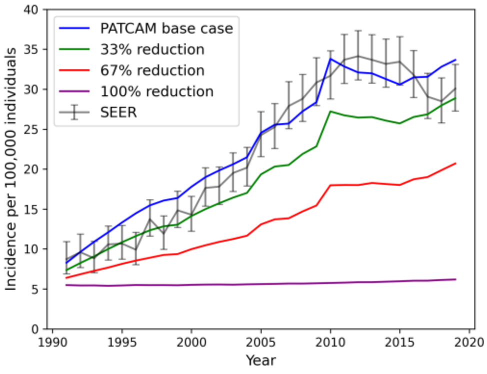

D)

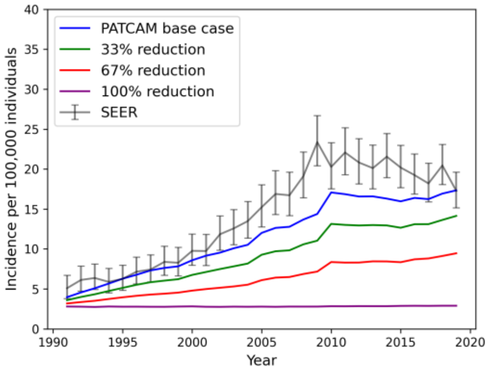

E)

F)

© 2026 Francis DO et al. *JAMA Network Open*

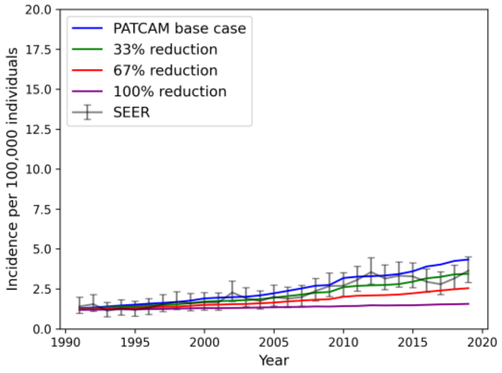

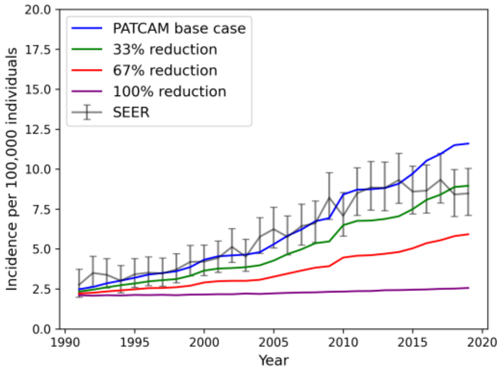

G)

H)

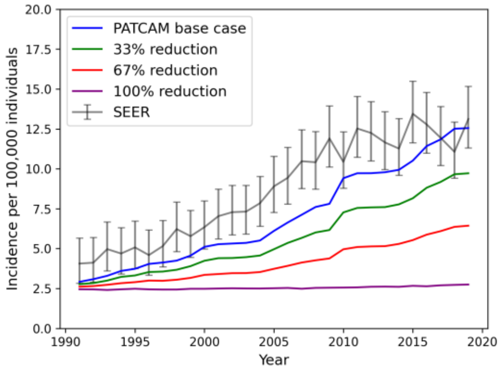

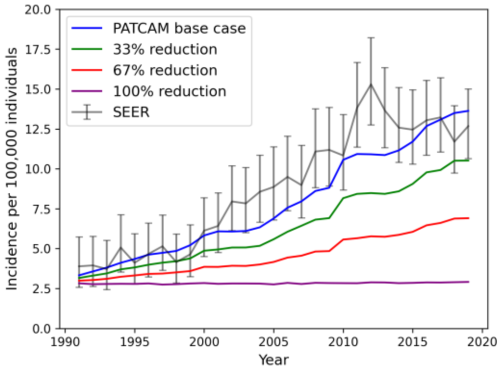

© 2026 Francis DO et al. *JAMA Network Open*

eFigure 7. Impact of reduction in thyroid ultrasound referral rates on thyroid cancer mortality.
A) Mortality for women aged 15-34; B) Mortality for women aged 35-49; C) Mortality for women aged 50-64; D) Mortality for women aged 65+; E) Mortality for men aged 15-34; F) Mortality for men aged 35-49; G) Mortality for men aged 50-64; H) Mortality for men aged 65+.

A)

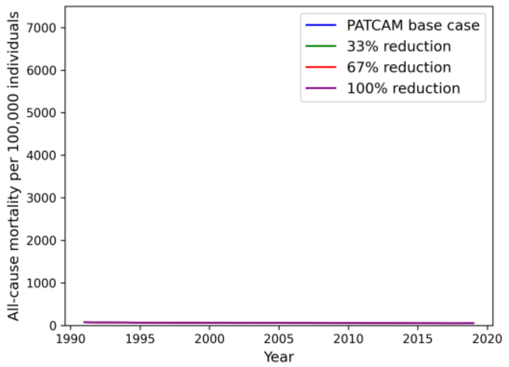

B)

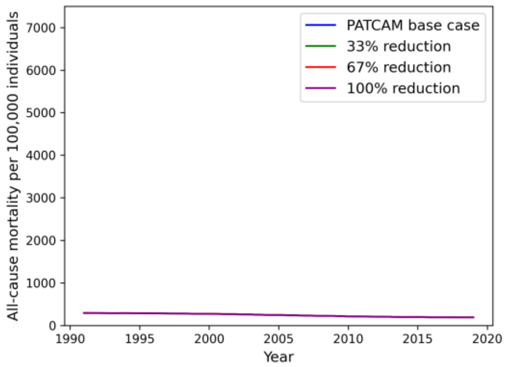

C)

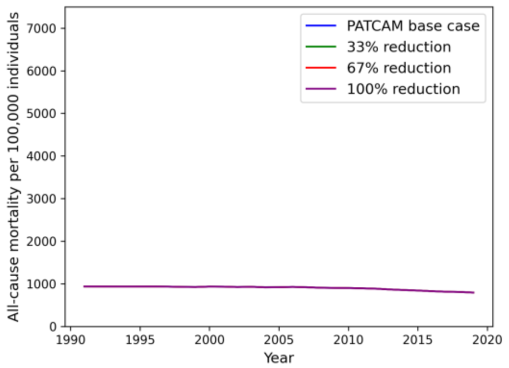

D)

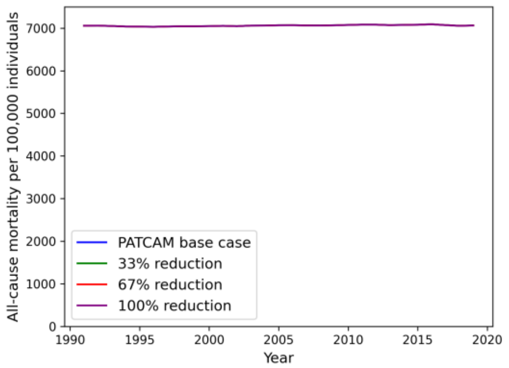

E)

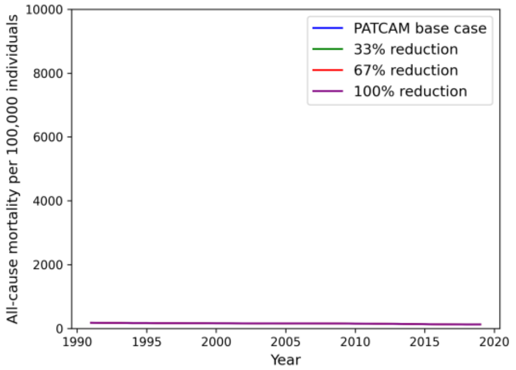

F)

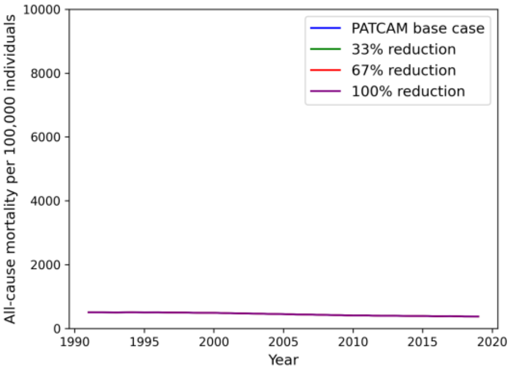

© 2026 Francis DO et al. *JAMA Network Open*

G)

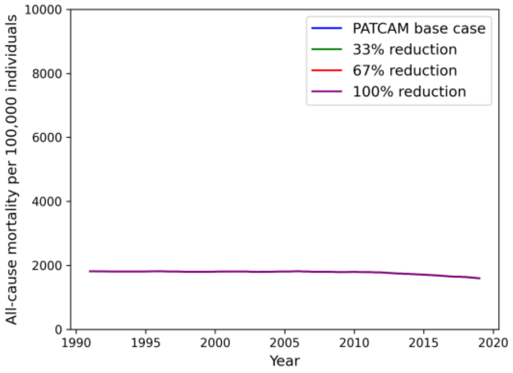

H)

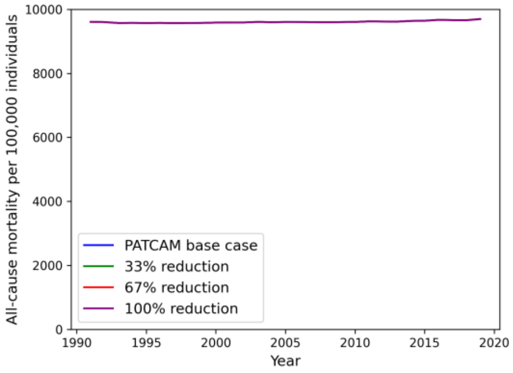

© 2026 Francis DO et al. *JAMA Network Open*

eReferences

1. US Census Bureau. Explore Census Data. (https://www.census.gov/).
2. Human Mortality Database. HMD USA Total Population. (https://www.mortality.org/).
3. Reiners C, Wegscheider K, Schicha H, et al. Prevalence of thyroid disorders in the working population of Germany: ultrasonography screening in 96,278 unselected employees. Thyroid 2004;14(11):926-932. DOI: 10.1089/thy.2004.14.926.
4. Hsiao V, Arroyo N, Fernandes-Taylor S, Chiu AS, Davies L, Francis DO. Letter to the Editor: sensitivity of palpation for detection of thyroid nodules with attention to size. Thyroid 2022;32(5):599-601. DOI: 10.1089/thy.2021.0692.
5. Hsiao V, Massoud E, Jensen C, et al. Diagnostic accuracy of fine-needle biopsy in the detection of thyroid malignancy: a systematic review and meta-analysis. JAMA Surg 2022;157(12):1105-1113. DOI: 10.1001/jamasurg.2022.4989.
6. National Cancer Institute. Surveillance, SEER*Stat Database: Incidence - SEER Research Data, 9 Registries; 2020 Sub (1975-2018) - Linked To County Attributes - Time Dependent (1990-2018) Income/Rurality Counties, National Cancer Institute, DCCPS, Surveillance Research Program, released April 2021, based on the. 2020 Submission November; 2023. Epidemiology, and End Results (SEER) Program:1969-2019. (https:// seer.cancer.gov/data/citation.html).

© 2026 Francis DO et al. *JAMA Network Open*
