# Supplementary material for: Overdiagnosis of Papillary Thyroid Cancer
Source: JAMA Netw Open. 2026 Feb 24;9(2):e2559852. doi: 10.1001/jamanetworkopen.2025.59852 (PMC12933285; doi:10.1001/jamanetworkopen.2025.59852)
Supplement: Supplement 2. — Data Sharing Statement [file jamanetwopen-e2559852-s002.pdf]

Data Sharing Statement

Francis. Overdiagnosis of Papillary Thyroid Cancer. *JAMA Netw Open*. Published February 24, 2026. doi:10.1001/jamanetworkopen.2025.59852

Data
Data available: Yes
Data types: Data dictionary
How to access data: alagoz@engr.wisc.edu
When available: With publication

Supporting Documents
Document types: None

Additional Information
Who can access the data: Researchers whose proposed use of the data has been approved.
Types of analyses: Any purpose.
Mechanisms of data availability: With investigator support.
